# Functional characterisation of cytochrome P450s involved in insecticide resistance in major malaria vector *Anopheles funestus* across Africa

**DOI:** 10.1186/1475-2875-13-S1-P46

**Published:** 2014-09-22

**Authors:** Charles Wondji, Mark Paine, Jacob Miranda, Sulaiman Ibrahim

**Affiliations:** 1 Vector Biology Department, Liverpool School of Tropical Medicine, Liverpool/Merseyside, UK

## 

Control of malaria relies heavily on the use of insecticides and resistance in malaria vectors such as *An. funestus*, is a serious threat to the success of these control tools.

Recent studies have shown that the duplicated cytochrome P450s, CYP6P9a and CYP6P9b shown to confer pyrethroid resistance, vary extensively in their allelic distributions across Africa and this is possibly impacting distribution of pyrethroid resistance in this specie.

Polymorphism in CYP6P9a and b in resistant mosquitoes from Benin, Uganda, Malawi, Mozambique and susceptible strain (FANG) was mapped. Models of these genes were constructed using CYP3A4-1TQN and 3D insecticides docked using GOLD. Membranes from these genes co-expressed with CPR were screened with fluorescent probes. Kinetic parameters and IC_50_ against ten insecticides were established with the probe diethoxyfluorescein. Metabolism assay was carried out against pyrethroids; time- and NADPH-dependent depletion and kinetic parameters determined. Site-directed mutagenesis was employed to change some key amino acids from resistant individuals to what is obtainable in the susceptible individuals, proteins expressed and metabolism assays carried out.

Docking results predict higher activity in alleles from Southern Africa compared with those from other regions. FANG alleles have the lowest activity. With the exception of FANG, the membranes metabolise diethoxyfluorescein very well, with the lowest Km and highest Vmax obtained from the Southern African alleles. Low IC_50_was obtained with pyrethroid and high IC_50_ with carbamates and DDT. The membranes metabolise type I and II pyrethroids with resistant alleles from Southern Africa having the highest activity, consistent with the highest resistance level to pyrethroids in this region. The susceptible strain is incapable of significant metabolism against pyrethroids tested. Metabolism assays using the mutants of CYP6P9 and b identified mutants incapable of appreciable metabolism of permethrin and deltamethrin.

The significant differences observed in the activity of different alleles of CYP6P9a and b probably explain the variation in distribution of pyrethroids resistance in Africa. Kinetic analysis is being carried out to establish steady state parameters using the proteins from the wild type and the mutants against pyrethroids. It’s hoped that these results will pinpoint the causative mutations responsible for resistance pattern with pyrethroids so that diagnostic tools could be established to easily detect this kind of resistance.

